# First record of the genus *Arabelia* Bosselaers, 2009 from China, with description of one new species (Araneae, Liocranidae)

**DOI:** 10.3897/BDJ.10.e85436

**Published:** 2022-05-10

**Authors:** Yannan Mu, Feng Zhang

**Affiliations:** 1 The Key Laboratory of Zoological Systematics and Application, Institute of Life Science and Green Development, Hebei University, Baoding, China The Key Laboratory of Zoological Systematics and Application, Institute of Life Science and Green Development, Hebei University Baoding China

**Keywords:** Morphology, taxonomy, habitus, biology

## Abstract

**Background:**

The spider family Liocranidae Simon, 1897 contains 35 genera and 308 species, including six genera and 33 species reported in China, which are: *Agroeca* Westring, 1861 (13 species), *Jacaena* Thorell, 1897 (7 species), *Mesiotelus* Simon, 1897 (1 species), *Oedignatha* Thorell, 1881 (2 species), *Paratus* Simon, 1898 (4 species), *Sesieutes* Simon, 1897 (1 species) and *Sphingius* Thorell, 1890 (5 species).

**New information:**

The spider genus *Arabelia* Bosselaers, 2009 is described from China for the first time, with one new species *Arabeliaxizang* sp. nov.

## Introduction

*Arabelia*, established by Bosselaers (2009), based on the female holotype *Arabeliapheidoleicomes* from Greece, is placed in Liocranidae by having a flat carapace, narrow eye field, lack of abdominal sclerotisation and the presence of an anterior epigynal hood. Bosmans (2011) first described the male of the type species, but placed it in the family Corinnidae (World Spider Catalog 2022). Here, we place this genus *Arabelia* in Liocranidae, based on following characters: 1) flat carapace; 2) narrow eye area, less than half carapace width and two eye rows nearly straight; 3) an anterior epigynal hood like *Mesiotelus* and 4) simple male palp like *Drassinella*. When we studied the specimens from Xizang of China, we indentified a new species belonging to this genus, based on the following combination of characters: 1) a pair of large COs; 2) an anterior hood; 3) anterior ST2 and posterior ST1 and 4) the oval-shaped bulb without apophysis and the sperm duct tapering from retrolateral to prolateral.

## Materials and methods

All measurements in the text are given in millimetres. The measurements of the legs are shown as total length (femur, patella, tibia, metatarsus, tarsus). Epigynes were removed and cleared in a pancreatin solution (Álvarez-Padilla and Hormiga 2007). All specimens are preserved in 75% alcohol. Photographs were taken using a Leica M205A stereomicroscope, equipped with a DFC 550 CCD camera. All specimens studied are deposited in the Museum of Hebei University (MHBU), Baoding, China.

The following abbreviations are used: AER—anterior eye row; ALE—anterior lateral eye; AME—anterior median eye; MOA—median ocular area; MS—median septum; PER—posterior eye row; PLE—posterior lateral eye; PME—posterior median eye; RTA—retrolateral tibial apophysis. Spination: d—dorsal; b—base; pl—prolateral; pv—proventral; rv—retroventral; v—ventral.

## Taxon treatments

### Arabelia xizang

Mu & Zhang, 2022 sp. n.

691183B7-CB26-55A3-8D2E-E08FA6F6BCA1

D096F18B-8FDC-4E4E-BCE1-A6DF4931261D

#### Materials

- **Type status:** Holotype. **Occurrence:** individualCount: 1; sex: male; lifeStage: adult; **Taxon:** scientificName: *Arabeliaxizang*; order: Araneae; family: Liocranidae; genus: Arabelia; **Location:** country: China; stateProvince: Xizang Autonomous Region; county: Markam; locality: between 3351-3352 kilometres of National Highway 318; verbatimElevation: 3127; verbatimLatitude: 29°44'19.18"N; verbatimLongitude: 98°49'33.57"E; **Event:** year: 2020; month: 7; day: 21; **Record Level:** institutionID: the Museum of Hebei University; institutionCode: MHBU
- **Type status:** Paratype. **Occurrence:** individualCount: 4; sex: female; lifeStage: adult; **Taxon:** scientificName: *Arabeliaxizang*; order: Araneae; family: Liocranidae; genus: Arabelia; **Location:** country: China; stateProvince: Xizang Autonomous Region; county: Markam; locality: between 3351-3352 kilometres of National Highway 318; verbatimElevation: 3127; verbatimLatitude: 29°44'19.18"N; verbatimLongitude: 98°49'33.57"E; **Event:** year: 2020; month: 7; day: 21; **Record Level:** institutionID: the Museum of Hebei University; institutionCode: MHBU

#### Description

Male **(Holotype)**: total length 3.76, carapace 1.57 long, 1.42 wide (CW); abdomen 2.19 long, 1.35 wide. Eye sizes and interdistances: AME 0.09, ALE 0.11, PME 0.08, PLE 0.09; AME–AME 0.04, AME–ALE 0.01, ALE–ALE 0.23, PME–PME 0.11, PME–PLE 0.06, PLE–PLE 0.36, ALE–PLE 0.06. Eye area 0.51 wide (EAW), cephalic region 0.78 wide (CRW), EAW/CRW 0.65. CRW/CW 0.55. MOA 0.22 long, anterior width 0.22, posterior width 0.27. Clypeal height 0.09 (CH), CH/AME 1.00. Chelicerae with three promarginal (largest at middle) and two retromarginal teeth (Fig. 3E). Labium 0.24 long, 0.27 wide. Sternum 0.97 long, 0.93 wide. Carapace nearly round, deep brown, with darker longitudinal markings either side of fovea. Fovea distinct, longitudinal. Abdomen grey, lacking dorsal scutum, covered with black hairs, two white patterns at middle part (Fig. 2A–C). Measurement and spines of legs as in Tables 1, 2.

Palp as in Fig. 3 (A–D). Femur longer than tibia and cymbium, femoral apophysis absent, ventrally with low hump at distal part (Fig. 3A–B). Tibia shorter than cymbium (about 0.6 times). RTA thin and short, hook-shaped (Fig. 3B–D). Cymbium two times longer than wide. Bulb narrower than width of cymbium. Tegulum oval, tegular apophysis absent. Sperm duct long, tapering from retrolateral to prolateral before entering base of embolus (Fig. 3D). Embolus thin and short, directed anteriorly. Conductor membranous, originating in middle of tegulum.

Female: One paratype total length 3.95, carapace 1.36 long, 1.24 wide (CW); abdomen 2.59 long, 1.50 wide. Eye sizes and interdistances: AME 0.08, ALE 0.09, PME 0.06, PLE 0.08; AME–AME 0.05, AME–ALE 0.01, ALE–ALE 0.20, PME–PME 0.09, PME–PLE 0.06, PLE–PLE 0.34, ALE–PLE 0.05. Eye area 0.44 wide (EAW), cephalic region 0.61 wide (CRW), EAW/CRW 0.72. CRW/CW 0.49. MOA 0.22 long, anterior width 0.19, posterior width 0.23. Clypeal height 0.07 (CH), CH/AME 0.88. Other characters as in male, except the slightly larger body size (Fig. 2D–E). Measurement and spines of legs as in Tables 1, 2.

Epigyne as in Fig. 4. Epigynal plate 1.3 times wider than long. MS wide. Semicircular hood located above MS (Fig. 4A, C). Copulatory openings large, oblique, anteriorly, separated about two times MS width. Copulatory ducts short and thick. Small, globular ST2 located at middle parts, kidney-shaped ST1 posterior (Fig. 4B). Fertilisation ducts downwards, located posteriorly of spermathecae (Fig. 4B, D).

#### Diagnosis

This new species is similar to the type species *A.pheidoleicomes* Bosselaers, 2009 in having similar-shaped palp in the male, the position of copulatory openings and the presence of an anterior hood in the female. However, it can be distinguished from *A.pheidoleicomes* Bosselaers, 2009 by: 1) the hook-shaped RTA (vs. straight, compare Fig. 3C and Fig. 15 in Bosmans 2011); 2) the slightly curved embolus(vs. straight, compare Fig. 3D and Fig. 16 in Bosmans 2011), 3) a large, wide and sclerotised hood (vs. small, compare Fig. 4A and Fig. 7F in Bosselaers 2009) and 4) the kidney-shaped spermathecae (vs. round, compare Fig. 4B and Fig. 7E in Bosselaers 2009).

#### Etymology

The specific name is derived from the type locality; noun.

#### Distribution

Known only from the type locality (China: Xizang).

#### Notes

**Figure 1.**
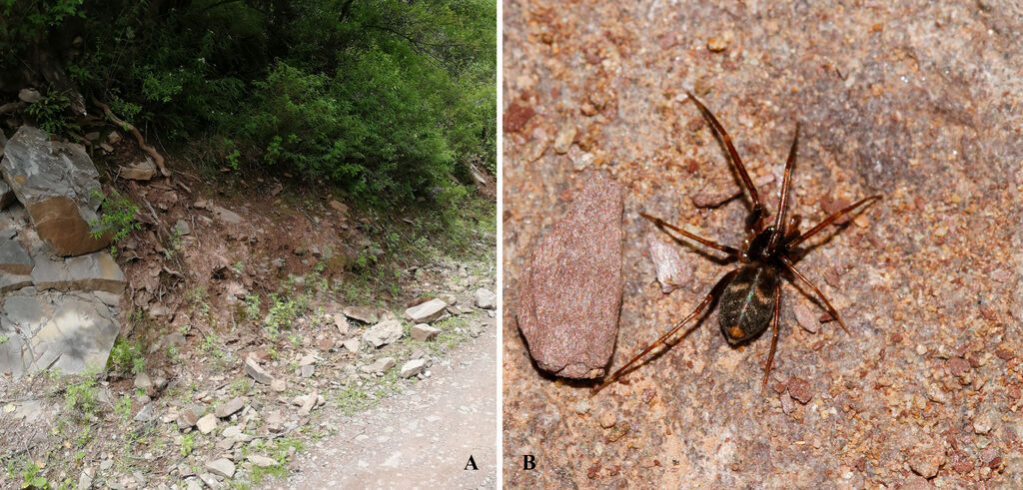
Habitat and female of *Arabeliaxizang* sp.nov.. **A** habitat; **B** living image (Photograph by Yannan Mu).

**Figure 2.**
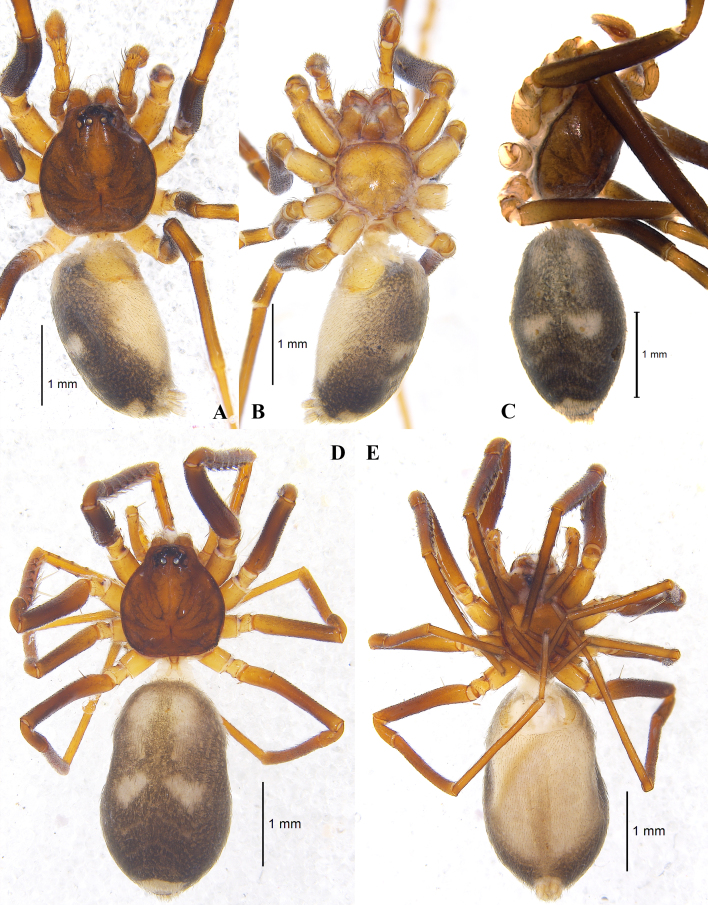
Habitus of *Arabeliaxizang* sp. nov.. **A** Male (holotype), dorsal view; **B** same, ventral view; **C** same, abdomen dorsal view; **D** Female (paratype), dorsal view; **E** same, ventral view.

**Figure 3.**
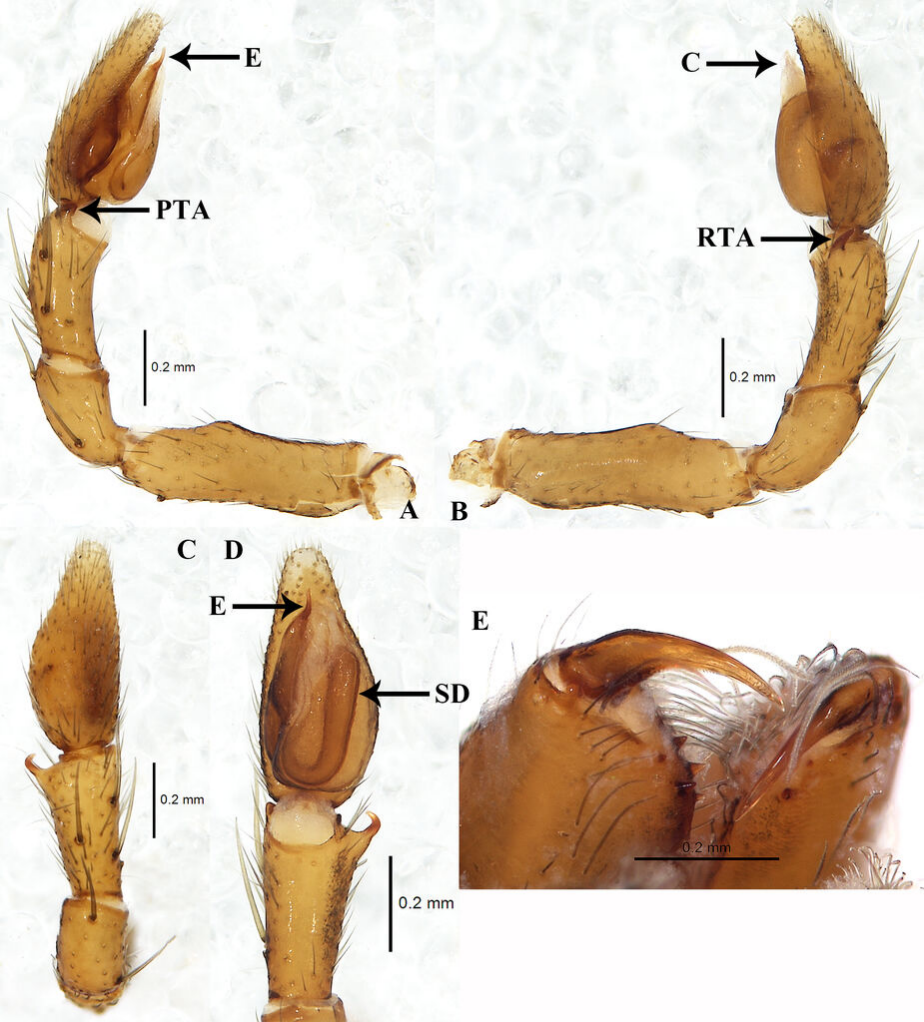
Male palp and chelicerae. **A** left palp, prolateral view; **B** same, retrolateral view; **C** Same, dorsal view; **D** same, ventral view; **E** chelicerae, retrolateral view. Abbreviations: C — conductor; E — embolus; PTA — prolateral tibial apophysis; RTA — retrolateral tibial apophysis; SD — sperm duct.

**Figure 4.**
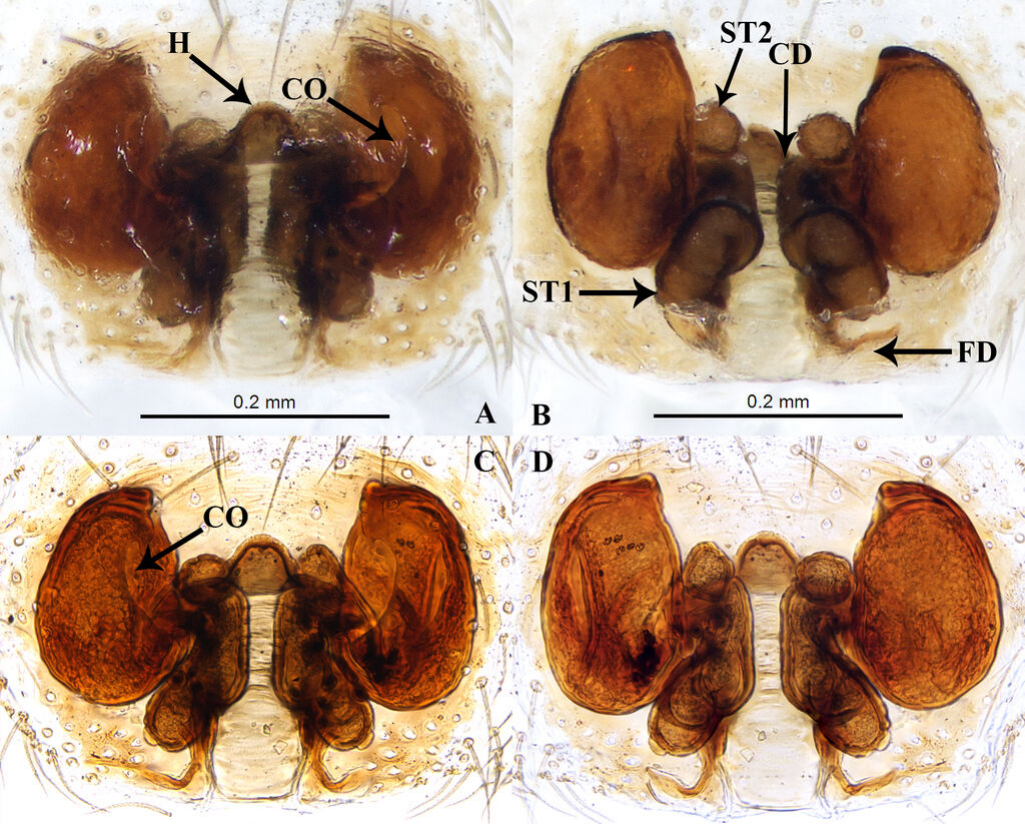
Female genitalia. **A** Epigyne, ventral view; **B** Vulva, dorsal view; **C** Epigyne, ventral view; **D** Vulva, dorsal view. (**A, B**: cleared in pancreatin solution; **C, D**: macerated in Holly Oil). Abbreviations: CO — copulatory opening; CD — copulatory duct; FD — fertilisation duct; H — hood; ST1 — primary spermatheca, ST2 — secondary spermatheca.

The spiders of this new species are found under stones on the side of roads (Fig. 1A). Female matured about half a month earlier than male (Fig. 1B). We collected the adult females and subadult male, the male description being based on the adult reared from the subadult.

According to Bosselaers (2009), the type species of *Arabelia* show clear myrmecophily, but we did not find ants or termites when collecting this new species under gravel. Moreover, there are also differences in the colour between *A.pheidoleicomes* and *A.xizang* **sp. n.** in the natural state and in alcohol. The former species has brown carapace, grey abdomen in the natural state and yellow carapace, white abdomen in alcohol, while the new species is black in the natural state and has brown carapace and grey abdomen in alcohol. However, due to its similar genital characters, we placed this new species in *Arabelia*.

## Supplementary Material

XML Treatment for Arabelia xizang

## Figures and Tables

**Table 1.** Measurement of legs:

m/f	Fe	Pa	Ti	Me	Ta	Total
Leg Ⅰ	2.70/1.79	0.93/0.68	2.98/1.88	2.70/1.72	1.41/0.90	10.67/6.97
Leg Ⅱ	1.94/1.47	0.78/0.59	1.97/1.44	1.80/1.29	0.95/0.74	7.44/5.53
Leg Ⅲ	1.46/1.19	0.56/0.49	1.32/1.05	1.41/1.11	0.82/0.70	5.57/4.54
Leg Ⅳ	2.03/1.68	0.70/0.55	1.89/1.62	2.03/1.74	1.02/0.87	7.67/6.46

**Table 2.** Spination of legs:

		Fe	Pa	Ti	Me	Ta
male	Leg Ⅰ	d 1 pl 2	–	pv 8 rv 9	pv 6 rv 6	–
	Leg Ⅱ	d 1	–	pv 7 rv 7	pv 5 rv 5	–
	Leg Ⅲ	d 1	–	–	–	–
	Leg Ⅳ	d 1	–	–	–	–
female	Leg Ⅰ	d 1 pl 2	–	pv 7 rv 7	pv 5 rv 5	–
	Leg Ⅱ	d 1	–	pv 7 rv 7	pv 5 rv 4	–
	Leg Ⅲ	d 1	–	–	–	–
	Leg Ⅳ	d 1	–	–	–	–
